# Vitamin D Analogues and Fracture Risk in Chronic Kidney Disease: A Systematic Review and Meta‐Analysis of Randomized Controlled Trials

**DOI:** 10.1002/jbm4.10611

**Published:** 2022-02-21

**Authors:** Nada Khelifi, Louis‐Charles Desbiens, Aboubacar Sidibé, Fabrice Mac‐Way

**Affiliations:** ^1^ CHU de Québec Research Center, Division of Nephrology Endocrinology and Nephrology Axis Quebec City Canada; ^2^ Faculty and Department of Medicine Université Laval Quebec City Canada; ^3^ Faculty of Medicine, Department of Social and Preventive Medicine Université Laval Quebec City Canada

**Keywords:** CHRONIC KIDNEY DISEASE, FRACTURE, RANDOMIZED CONTROLLED TRIALS, SYSTEMATIC REVIEW AND META‐ANALYSIS, VITAMIN D ANALOGUES

## Abstract

Vitamin D receptor agonists (VDRAs) are commonly prescribed in chronic kidney disease (CKD). However, their protective effects on bone remain controversial. We performed a systematic review and meta‐analysis of randomized controlled trials (RCTs) to evaluate the effect of VDRAs on fracture risk and bone mineral density (BMD) in adult patients with CKD. We searched MEDLINE, EMBASE, CENTRAL, ClinicalTrials.gov, and the WHO's International Clinical Trials Registry Platform databases from inception to June 19, 2021. We included RCTs comparing VDRAs, to placebo or another medication, in adults with CKD requiring or not dialysis. Conference abstracts and trials involving kidney transplant recipients and/or comparing VDRAs to antiresorptive or anabolic bone therapy were excluded. Primary outcome was fracture at any anatomical site. Secondary outcomes were BMD at femoral neck, lumbar spine, and/or total hip. Prespecified subgroup analyses were conducted according to baseline demographics, overall risk of bias, and follow‐up time. From 6868 references retrieved, eight RCTs were eligible: five reported fracture, two reported BMD, and one reported both outcomes. As comparator, one study used no VDRAs, one used nutritional intervention and no medication, and six used placebo. In meta‐analysis, VDRAs were not associated with a significant reduction in total fractures in overall (risk ratio = 0.79, 95% confidence interval 0.38–1.65, I^2^ = 0%, six trials, 1507 participants, 27 fractures) or in prespecified subgroup analyses. Three trials reported BMD at different sites and with different BMD measurements; thus, a meta‐analysis could not be performed. Two RCTs were at high risk of bias, notably because of deviations from the intended interventions. As limitation, we have to mention the low total number of fractures included in our meta‐analysis. In conclusion, current evidence from RCTs is insufficient to associate VDRAs with bone protection in CKD. Further large and long‐term studies specifically designed to evaluate the efficacy of VDRAs on bone outcomes are thus required. © 2022 The Authors. *JBMR Plus* published by Wiley Periodicals LLC on behalf of American Society for Bone and Mineral Research.

## 1 Introduction

Chronic kidney disease (CKD) is a well‐recognized public health care issue affecting approximately 700 million people worldwide.^(^ ^1^ ^)^ CKD is associated with imbalance in mineral metabolism, vascular calcification, and bone remodeling described as the CKD‐mineral and bone disorders (CKD‐MBD).^(^ ^2^ ^)^ As kidney function declines, reduced renal hydroxylation of vitamin D impairs 1,25(OH)2D synthesis.^(^ ^3^, ^4^ ^)^ Declining levels of 1,25(OH)2D leads to hypocalcemia and sustained elevation of parathyroid hormone (PTH) levels, which are associated with bone loss and higher risk of fracture among individuals suffering from CKD.^(^ ^5^, ^6^, ^7^, ^8^, ^9^, ^10^ ^)^ Indeed, individuals with end‐stage CKD have a 4‐ to 17‐fold increase in fracture risk compared with the general population.^(^ ^11^, ^12^ ^)^

Treatment for these CKD mineral abnormalities include “active” vitamin D or vitamin D receptor analogues (VDRAs) as they partially restore mineral homeostasis^(^ ^13^, ^14^ ^)^ in order to decrease the bone consequences of hyperparathyroidism.^(^ ^15^, ^16^, ^17^ ^)^ Over the past decades, only few non‐randomized^(^ ^18^, ^19^, ^20^ ^)^ and randomized controlled trials (RCTs)^(^ ^21^ ^)^ have associated VDRA usage with increased bone mineral density (BMD) in CKD.^(^ ^18^, ^19^, ^20^, ^21^ ^)^ In 2009, two systematic reviews of RCTs comparing the effect of VDRAs to either placebo or no treatment on fracture risk and BMD in CKD have been conducted.^(^ ^22^, ^23^ ^)^ Unfortunately, data were insufficient to perform a meta‐analysis, thus limiting the ability to conclude on the effect of VDRAs on bone outcomes. Furthermore, VDRAs were proven efficient for reducing PTH levels, but their usage was also associated with increased blood phosphate and calcium levels.^(^ ^13^, ^22^, ^23^ ^)^ Some observational data also suggested a mortality benefit with the use of VDRAs in dialysis populations,^(^ ^24^, ^25^, ^26^, ^27^ ^)^ although this has been contested by other studies.^(^ ^28^ ^)^

Currently, evidence gaps demonstrating clear benefits on clinical endpoints such as fractures prevent firm recommendations regarding VDRA usage in current CKD‐MBD guidelines. As a result, clinicians are struggling to find the optimal management approach to adequately treat these patients. The primary objective of this systematic review was to evaluate the impact of VDRAs compared with placebo or other medications on fracture risk in RCTs of adult patients with CKD. The secondary objective was to evaluate the effect of VDRAs on BMD at femoral neck, lumbar spine, and total hip.

## 2 Materials and Methods

This review was conducted according to the Cochrane Handbook for Systematic Reviews of Interventions.^(^ ^29^ ^)^ Reporting was consistent with the Preferred Reporting Items for Systematic Review and Meta‐Analysis (PRISMA) statement.^(^ ^30^ ^)^ The protocol was registered in the Prospective Register of Systematic Reviews (PROSPERO) (CRD42020154915).

### 2.1 Data sources and search strategy

MEDLINE, EMBASE, CENTRAL, ClinicalTrials.gov, and the WHO's International Clinical Trials Registry Platform (ICTRP) searches were completed from inception to June 19, 2021, using individualized search strategies. The search strategy was composed of free text and indexed terms (MeSH, Emtree) related to the population (chronic kidney disease) and the intervention (VDRAs).^(^ ^22^, ^23^, ^31^ ^)^ Filters were used for the type of study (randomized controlled trials) using a sensitivity‐maximizing filter for MEDLINE^(^ ^29^, ^32^ ^)^ and a sensitivity maximizing filter with increased specificity for EMBASE.^(^ ^33^ ^)^ Terms related to outcomes (fractures and BMD) were not included in the search strategy. References from included articles were manually checked for other potentially relevant literature. Search strategies for each electronic database are displayed in Supplemental Appendix S1.

### 2.2 Eligibility criteria

We included RCTs that studied adults ≥18 years (at least 80% of the study population) with stage 3 to 5 CKD or dialysis^(^ ^34^ ^)^ and comparing VDRAs, alone or in combination, with placebo or another medication. Studies using natural forms of vitamin D (such as cholecalciferol and ergocalciferol) either in the control group or in both groups were eligible. Studies were required to report at least one of the following outcomes: fracture occurring at any site (as prespecified outcomes, adverse events, or post hoc analysis) or BMD at femoral neck, lumbar spine, or total hip. Conference abstracts and studies comparing VDRAs to either antiresorptive agents (bisphosphonates, denosumab, raloxifene) or anabolic bone therapies (teriparatide) were excluded. Studies involving kidney transplant recipients were also excluded. Neither language nor publication's year restriction was applied.

### 2.3 Data management and selection process

Results from the search strategies were merged and duplicates were removed using EndNote X9 (Thomson Reuters, New York, NY, USA). Unique references were then exported to Excel software (Microsoft Corporation, Redmond, WA, USA). Two reviewers (NK, LCD) independently first assessed the eligibility of studies by title and abstract. To maximize screening sensitivity, outcomes (fracture or BMD) were not used as inclusion criteria in the first screening. Then, full texts of potentially eligible articles were assessed for all inclusion criteria by two reviewers (NK, LCD). Disagreements were resolved through discussion. Studies written in other language than French or English were translated before assessment by linguists preferably or using Google Translate (Google LLC, Mountain View, CA, USA).^(^ ^35^ ^)^

### 2.4 Data extraction

Data extraction was performed by two independent reviewers (NK, LCD) using an abstraction form pilot‐tested beforehand for completeness and clarity. When multiple reports of the same study were identified, the report containing the most complete information for the review was included. Other reports were used to gather additional data when necessary.

For each included study, methodological parameters (study design, setting, details of random sequence generation, follow‐up period, prespecified outcomes, and funding), characteristics of the study population (sample size, age, sex, stage of CKD, baseline estimated glomerular filtration rate, comorbidities), description of intervention(s) and comparator(s) (medication, routes of delivery, doses, length of treatment), outcomes (fracture and BMD sites, measurement methods, BMD units), and effects of interventions (loss to follow‐up in each arm, number of fractured patients, obtained BMD) were collected. When possible, fracture events and BMD measurements were recorded for the longest follow‐up duration. When both absolute (g/cm^2^) and relative change from baseline (%) for BMD measurements were provided, absolute values were preferably collected. Authors were contacted when additional information was needed.

### 2.5 Risk of bias and quality assessment

Two independent reviewers (NK, LCD) used the revised version of Cochrane Risk‐of‐Bias Tool (RoB2) for randomized trials^(^ ^32^ ^)^ to assess the methodological quality of included studies. If one or more individual domains were assessed as having a high risk of bias, the trial was rated as having a high risk of bias. Disagreement between reviewers was solved through discussion.

### 2.6 Statistical analysis and data synthesis

The Review Manager Software version 5.4 (The Cochrane Collaboration, Copenhagen, 2014) was used to carry out the analysis. Meta‐analysis was conducted when at least three studies were available by outcome of interest. When data extraction could not be performed, studies were used as qualitative analysis. Fractures were pooled as risk ratios (RR) using the Mantel–Hantzel method with random effect models. BMD was pooled as mean differences (MD) using the inverse variance method with random effect models.^(^ ^36^ ^)^ Furthermore, BMD measurements at different anatomical sites and/or expressed with different units were pooled separately. BMD expressed with standard error of the mean (SEM) were transformed to standard deviation (SD) using formulas provided in the Cochrane Handbook.^(^ ^29^ ^)^ We preferably used trial outcomes and summary effect measures based on intention‐to‐treat data. All estimates were presented with 95% confidence intervals.

### 2.7 Additional analysis

Statistical heterogeneity was assessed with the Cochrane's I^2^ statistic.^(^ ^37^ ^)^ Heterogeneity was defined as negligible (0%–40%), moderate (30%–60%), substantial (50%–90%), and considerable (75%–100%).^(^ ^37^ ^)^ A priori specified subgroup analyses were performed for the age (<50, 50 to 65, >65 years old), sex (<25%, 25–75%, >75% male), dialysis status (no dialysis, dialysis), baseline PTH serum level (<150, 150–299, 300–600, >600 pg/mL), follow‐up period (<1 or ≥ 1 year), and overall risk of bias (low, high, and some concerns). The risk of publication bias was assessed by visual evaluation of funnel plots.

## 3 Results

**Fig. 1:**
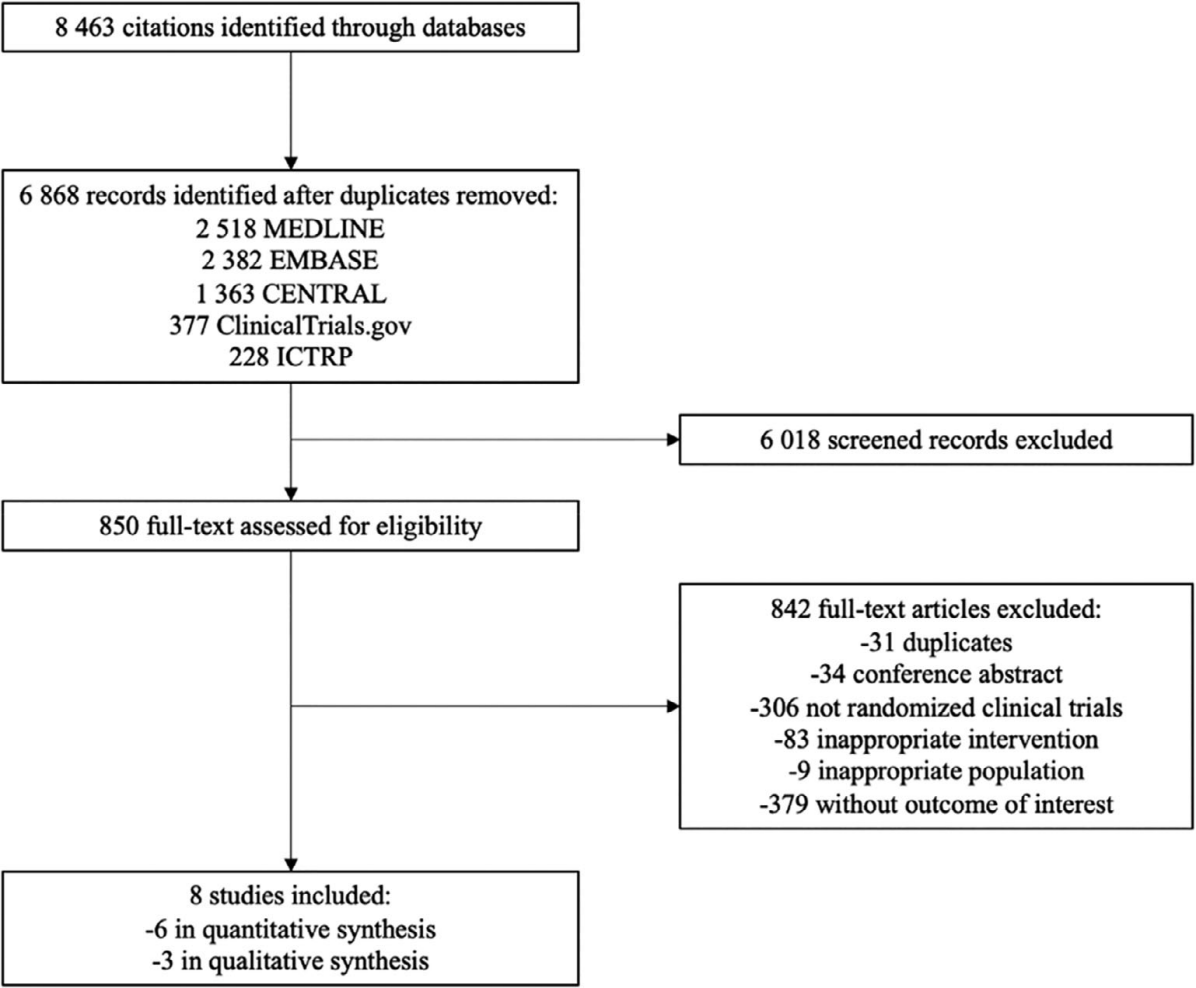
PRISMA flow diagram of study selection. PRISMA = Preferred Reporting Items for Systematic Reviews and Meta‐analyses.

From 6868 unique citations retrieved through MEDLINE, EMBASE, CENTRAL, ClinicalTrials.gov, and the WHO's ICTRP databases, full‐text screening was required for 850 and eight^(^ ^21^, ^38^, ^39^, ^40^, ^41^, ^42^, ^43^, ^44^ ^)^ studies met inclusion criteria for data extraction (Fig. 1). All studies were published except one^(^ ^42^ ^)^ trial that was completed in the WHO's ICTRP.

### 3.1 Characteristics of included studies

Study characteristics of the eight included trials are presented in Table 1. All included studies were parallel‐group RCTs.^(^ ^21^, ^38^, ^39^, ^40^, ^41^, ^42^, ^43^, ^44^ ^)^ The sample size varied from 26 to 976 participants, for a total of 1610 subjects. Mean age ranged from 42 to 65 years. Only one^(^ ^39^ ^)^ study did not report data on sex, whereas the other ones^(^ ^21^, ^38^, ^40^, ^41^, ^42^, ^43^, ^44^ ^)^ included both men and women. Four 21, 38, 40, 42 ^)^ included adult patients with CKD stages 3 to 5, three 39, 41, 43 ^)^ were exclusively conducted on chronic hemodialysis patients, while another one^(^ ^44^ ^)^ was exclusively performed in continuous ambulatory peritoneal dialysis (CAPD) patients. Interventions included calcitriol (0.25–1 μg/d),^(^ ^38^, ^39^, ^42^ ^)^ alfacalcidol (0.5 μg/d),^(^ ^21^, ^41^, ^44^ ^)^ paricalcitol (2 μg/d),^(^ ^40^ ^)^ and 2‐methylene‐19‐nor‐20S‐1α,25‐dihydroxyvitamin D3 (DP001 at 1 750 ng/d).^(^ ^43^ ^)^ As comparator, one^(^ ^44^ ^)^ study used nutritional intervention, two^(^ ^41^, ^44^ ^)^ studies used no medication, and six^(^ ^21^, ^38^, ^39^, ^40^, ^42^, ^43^ ^)^ studies used placebo. Five studies also allowed the use of PTH‐lowering therapies in intervention and comparator groups, including cinacalcet hydrochloride^(^ ^41^ ^)^ and phosphate binders^(^ ^21^, ^38^, ^39^, ^41^, ^43^ ^)^ (aluminium hydroxide, lanthanum carbonate, calcium carbonate, calcium acetate, and sevelamer hydrochloride). The exposure length varied from 12 weeks to 5 years and subjects from five^(^ ^21^, ^38^, ^39^, ^41^ ^)^ studies were exposed to VDRAs for ≥1 year. Of the eight^(^ ^21^, ^38^, ^39^, ^40^, ^41^, ^42^, ^43^, ^44^ ^)^ eligible studies, five^(^ ^39^, ^40^, ^41^, ^42^, ^43^ ^)^ reported fracture as an outcome or an adverse event (AE), two^(^ ^21^, ^44^ ^)^ reported BMD as an outcome, and one^(^ ^38^ ^)^ study reported both outcomes (fracture as AE and BMD).

**Table 1:** Characteristics of Included Studies

Study year (acronym)	Population	Age (years)^a^ age range	Male sex (%)	Baseline iPTH (pg/mL)^a^	Duration	Intervention			Comparator			Outcome
						Protocol (daily doses)	*n*	Obtained iPTH (pg/mL)^a^	Protocol (daily doses)	*n*	Obtained iPTH (pg/mL)^a^	
Baker, 1986^(^ ^39^ ^)^	ESRD on HD	42 (NR) 17–59 years	NR	Calcitriol: 180 (±36)^b^ Placebo: 250 (±37)^b^	5 years	Calcitriol 0.25 to 1 μg	38	40.0 (±1.0)^b^	Placebo	38	350.0 (±2.0)^b^	Fracture
Gnudi, 2010 (VDDT)^(^ ^42^ ^)^	eGFR 30 to 59 mL/min and T2DM	64.0 (±7.8) 40–75 years	70	61.6 (±34.0)	48 weeks	Calcitriol 0.25 μg	72	NR	Placebo	68	NR	Fracture (AE)
Thadhani, 2012 (PRIMO)^(^ ^40^ ^)^	eGFR 15 to 60 mL/min/1.73 m^2^	65.0 (±11.8) 31–90 years	70	104.5 (68.5–158.0)	48 weeks	Paricalcitol 2 μg	115	−83.13 (95% CI –97.81, −68.45)^c^	Placebo	112	+0.87 (95% CI –13.80, 15.53)^c^	Fracture (AE)
Thadhani, 2017^(^ ^43^ ^)^	ESRD on HD	58.7 (±12.0) 26–89 years	50	498.0 (±256.0)	12 weeks	DP001 1750 ng 3 times per week post dialysis	34	−45.0 (±28.0)^c^	Placebo 3 times per week post dialysis	28	+36.8 (±51.0)^c^	Fracture (AE)
Shoji, 2018 (J‐DAVID)^(^ ^41^ ^)^	ESRD on HD	63.5 (±10.0) 20–80 years	62	86.6 (46.8–130.0)	48 months	Alfacalcidol 0.5 μg	495	115.0 (60.0–190.0)	No VDRAs	481	120.0 (65.0–190.0)	Fracture (AE)
Przedlacki, 1995^(^ ^38^ ^)^	GFR ≤ 51.2 mL/min	49.8 (±10.4) 34–66 years	60	136.5 (±44.7)	12 months	Calcitriol 0.25 μg	13	105.8 (±48.0)	Placebo	13	151.4 (±43.4)	Fracture (AE) BMD FN, LS
Rix, 2004^(^ ^21^ ^)^	ClCr 10 to 60 mL/min	52.5 (NR) 35–72 years	69	152.0 (±258.0)	18 months	Alfacalcidol 0.25 to 0.75 μg	18	−27.0 (±9.0)^c^	Placebo	18	+53.0 (±20.0)^c^	BMD FN, LS
Son, 2006^(^ ^44^ ^)^	ESRD on CAPD	51.6 (±12.3) NR	54	107.8 (±109.4)	8 months	Alfacalcidol 0.5 μg	17	78.6 (±53.9)	No medication	23	119.1 (±164.0)	BMD FN, LS
									Nutritional education	27	54.0 (±68.2)	

eGFR = estimated glomerular filtration rate; ESRD = end‐stage renal disease; HD = hemodialysis; T2DM = type 2 diabetes mellitus; DP001 = 2‐methylene‐19‐nor‐20S‐1α,25‐dihydroxyvitamin D3; AE = adverse event; BMD = bone mineral density; PTH = parathyroid hormone; FN = femoral neck; LS = lumbar spine; NR = not reported; CAPD = continuous ambulatory peritoneal dialysis; P binders = phosphate binders; AL(OH)_3_ = aluminium hydroxide; CaCO_3_ = calcium carbonate; Nb = number of patients.

^a^ Age and iPTH are expressed in means (±SDs) or medians (25th–75th percentile) unless otherwise indicated.

^b^ Expressed as median (± ½ mid‐quartile range).

^c^ Percentage of change from baseline expressed as means (95% confidence intervals or ± SD).

### 3.2 Fractures

**Fig. 2:**
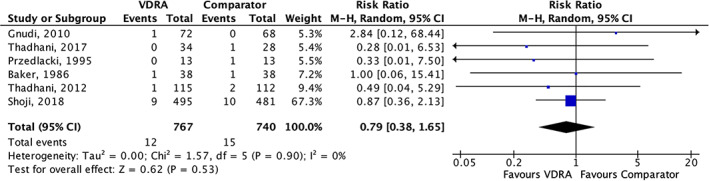
Forrest plot on the effects of vitamin D receptor agonist (VDRA) versus comparator on fracture risk at any anatomical site. Fractures at any anatomical sites were pooled as risk ratios (symbolized as blue boxes) with 95% confidence intervals (symbolized as black lines) from random effect models. The size of the box represents the weight attributed to each study in the meta‐analysis. CI = confidence interval; VDRA = vitamin D receptor agonist.

Six^(^ ^38^, ^39^, ^40^, ^41^, ^42^, ^43^ ^)^ studies including 1507 participants and reporting 27 fractures were pooled in a meta‐analysis. No significant effect of VDRAs on fracture risk compared with placebo or no VDRAs was observed (risk ratio [RR] = 0.79; 95% confidence interval [CI] 0.38–1.65; I^2^ = 0%; Fig. 2). As shown in Table 2, VDRAs were not associated with fracture risk compared with placebo or no VDRAs in any of the a priori specified subgroup analysis. No publication bias was detected for fractures on funnel plot (Supplemental Appendix S2).

**Table 2:** Subgroup Analysis of VDRA Impact on Fracture Risk

Subgroup	No. of studies	No. of patients	Risk ratio (95% CI)	I^2^
Mean age				
<50 years	2	102	0.62 (0.08, 4.84)	0%
50–65 years	4	1405	0.82 (0.38, 1.80)	0%
>65 years	0	0	NE	NE
Sex				
<25% males	4	1405	0.82 (0.38, 1.80)	0%
25–75% males	1	26	0.33 (0.01, 7.50)	NE
>75% males	0	0	NE	NE
Not reported	1	72	1.00 (0.07, 15.38)	NE
CKD type				
No dialysis	2	253	0.42 (0.06, 2.81)	0%
Dialysis	4	1254	0.88 (0.40, 1.96)	0%
Baseline PTH levels				
<150 pg/mL	4	1369	0.83 (0.38, 1.81)	0%
150–299 pg/ mL	1	76	1.00 (0.06, 15.41)	NE
300–600 pg/mL	1	62	0.28 (0.01, 6.53)	NE
>600 pg/mL	0	0	NE	NE
Follow‐up				
<1 year	4	429	0.67 (0.13, 3.41)	0%
>1 year	3	1078	0.83 (0.37, 1.88)	0%
Overall risk of bias				
Low	0	0	NE	NE
Some concerns	4	1405	0.82 (0.38, 1.80)	
High	2	102	0.62 (0.08, 4.84)	0%

VDRA = vitamin D receptor analogue; CI = confidence interval; NE = not estimable; CKD = chronic kidney disease; PTH = parathyroid hormone.

### 3.3 Bone mineral density

**Fig. 3:**
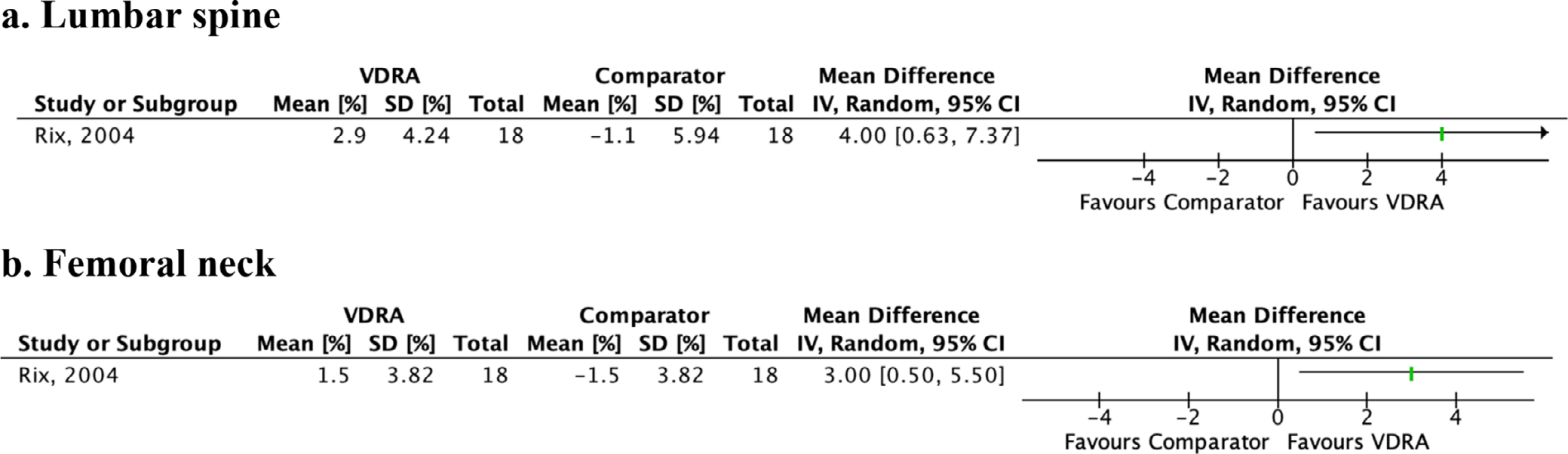
Effect of vitamin D receptor agonist (VDRA) versus comparator on percentage change from baseline in bone mineral density (%) for (*A*) lumbar spine and (*B*) femoral neck. Bone mineral density is presented as mean differences (symbolized as green lines) with 95% confidence intervals (symbolized as black lines). No meta‐analysis was conducted. CI = confidence interval; VDRA = vitamin D receptor agonist.

**Fig. 4:**
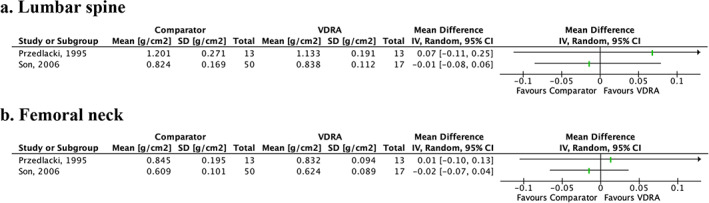
Effect of vitamin D receptor agonist (VDRA) versus comparator on percentage change from baseline in bone mineral density (%) for (*A*) lumbar spine and (*B*) femoral neck. Bone mineral density is presented as mean differences (symbolized as green lines) with 95% confidence intervals (symbolized as black lines). No meta‐analysis was conducted. CI = confidence interval; VDRA = vitamin D receptor agonist.

Three^(^ ^21^, ^38^, ^44^ ^)^ studies including 129 participants reported BMD at lumbar spine and femoral neck as an outcome, but no study reported BMD at total hip. Of these three studies, two^(^ ^38^, ^44^ ^)^ reported BMD as absolute density values (g/cm^2^) and one^(^ ^21^ ^)^ reported BMD as percentage of change from baseline. Because data were not sufficient, meta‐analysis could not be conducted. Only one^(^ ^21^ ^)^ study associated VDRA use with significant increase in BMD (percentage of change from baseline) compared with placebo at lumbar spine (mean difference [MD] = 4.00; 95% CI 0.63–7.37, Fig. 3*A*) and femoral neck (MD = 3.00; 95% CI 0.50–5.50], Fig. 3*B*). In the two other^(^ ^38^, ^44^ ^)^ studies, no significant differences, reported as absolute change from baseline (g/cm^2^), were found for lumbar spine (MDs = 0.07 [95% CI −0.11 to 0.25] and −0.01 [95% CI −0.08 to 0.06], Fig. 4*A*) and femoral neck (MDs = 0.01 [95% CI −0.10 to 0.13] and − 0.02 [95%CI −0.07, 0.04], Fig. 4*B*). Publication bias was not assessed for BMD because a meta‐analysis was not performed.

### 3.4 Risk of bias within studies

The risk‐of‐bias assessment results are presented in Tables 3 and 4. The overall risk of bias was considered “high” for two^(^ ^38^, ^39^ ^)^ studies because of notable baseline differences between groups. The six^(^ ^21^, ^40^, ^41^, ^42^, ^43^, ^44^ ^)^ other studies had “some concerns” because of important lost to follow‐up in studies, no blinding of outcome assessment, and fracture assessment was prone to information bias (measured as AE).

**Table 3:** Detailed Risk of Bias Assessment of Included Studies for Fracture

Study	Randomization process	Deviations from intended interventions	Missing outcome data	Outcome measurement	Selective reporting	Overall
Baker, 1986^(^ ^39^ ^)^	High	High	High	Low	Some concerns	High
Przedlacki, 1995^(^ ^38^ ^)^	High	Low	Low	Some concerns	Some concerns	High
Gnudi, 2010^(^ ^42^ ^)^	Some concerns	Low	Some concerns	Some concerns	Some concerns	Some concerns
Thadhani, 2012^(^ ^40^ ^)^	Low	Low	Some concerns	Some concerns	Some concerns	Some concerns
Thadhani, 2017^(^ ^43^ ^)^	Low	Low	Some concerns	Some concerns	Some concerns	Some concerns
Shoji, 2018^(^ ^41^ ^)^	Low	Low	Some concerns	Some concerns	Low	Some concerns

**Table 4:** Detailed Risk of Bias Assessment of Included Studies for Bone Mineral Density

Study	Randomization process	Deviations from intended interventions	Missing outcome data	Outcome measurement	Selective reporting	Overall
Przedlacki, 1995^(^ ^38^ ^)^	High	Low	Low	Low	Some concerns	High
Rix, 2004^(^ ^21^ ^)^	Low	Low	Low	Low	Some concerns	Some concerns
Son, 2006^(^ ^44^ ^)^	Low	Low	Some concerns	Some concerns	Some concerns	Some concerns

## 4 Discussion

In this systematic review, we assessed the impact of VDRAs on bone outcomes in adult patients with CKD. Although VDRAs were not associated with a significant effect on fracture risk, neither in overall nor in subgroup meta‐analyses, uncertainty remains high in these analyses. Because of insufficient and inconsistent data regarding BMD, a meta‐analysis could not be performed. Nevertheless, one^**(**^ ^**21**^ ^**)**^ included study reported significantly increased BMD with alfacalcidol at lumber spine and femoral neck sites.

Secondary hyperparathyroidism (SHPT) is a serious complication for CKD population. Phosphate retention together with 1,25(OH)2D deficiency are responsible for the progressive increases in PTH levels in CKD.^(^ ^45^ ^)^ Over time, sustained high PTH levels induce states of high bone turnover, contributing to vascular calcification, which defines the CKD‐MBD syndrome. Vascular calcification ultimately leads to arterial stiffening and cardiac hypertrophy and contributes to increasing cardiovascular events and mortality in CKD.^(^ ^46^, ^47^, ^48^ ^)^ Similarly, SHPT increases bone resorption, leading to reduced bone mass, and consequently, elevated fracture risk.^(^ ^49^ ^)^

The current management of CKD‐MBD relies on maintaining acceptable levels of mineral metabolism parameters in an attempt to prevent the development of SHPT. Among the available agents, VDRAs were proven effective in inhibiting PTH secretion by acting directly on the parathyroid gland.^(^ ^50^, ^51^ ^)^ The goal of VDRA therapy is therefore to minimize the bone consequences of hyperparathyroidism and to ultimately reduce fracture risk. However, excessive use of VDRAs in CKD has been associated with development of adynamic bone disease and vascular calcification.^(^ ^52^, ^53^, ^54^, ^55^ ^)^ The beneficial effect of VDRA treatment on bone outcomes is thus essential to understanding and guiding clinicians in the care of CKD patients.

Two^(^ ^22^, ^23^ ^)^ Cochrane Collaboration systematic reviews of RCTs evaluating the effect of VDRAs compared with either placebo or no treatment on fracture risk and BMD (only studies reporting BMD as absolute measurements were eligible) in CKD were conducted in 2009. However, authors were not able to perform a meta‐analysis owing to insufficient data. By performing this review, we were able to include six additional studies: four^(^ ^40^, ^41^, ^42^, ^43^ ^)^ that included fracture events and two that were conducted before Cochrane's reviews (one^(^ ^56^ ^)^ study reporting relative BMD change from baseline and one^(^ ^44^ ^)^ study using nutritional education as comparator). The key findings from our meta‐analysis are consistent with previous reviews in this area and suggest that current RCTs do not provide sufficient and precise evidence that VDRAs affect fracture risk and BMD in CKD.

Unfortunately, no large‐scale RCT has yet specifically investigated fracture risk, and several well‐designed studies with VDRAs have not reported fracture events. Thus, the event rates associated with the intervention and comparator may not reflect the true rates. The widespread clinical use of VDRAs within the CKD patient population contrasts with the inconsistent trials and lack of patient‐level outcome studies, which does not allow for appropriate evaluation of the impact of VDRAs on fracture risk. However, our meta‐analysis remains the most comprehensive and current assessment of RCTs that report incidence of fractures throughout a VDRA intervention in CKD patients.

Although our systematic review did not observe a beneficial effect of VDRAs on clinical bone outcomes, previous RCTs evaluating VDRAs on bone histomorphometry parameters have reported positive effects. Indeed, after 8 months of therapy with calcitriol, the mean bone‐formation rate decreased significantly in predialysis patients, while it increased in the placebo group.^(^ ^57^ ^)^ Approximately 25% of calcitriol‐treated patients developed adynamic bone disease though. Mineralization parameters and bone volume did not change significantly with calcitriol. In another study, treatment with alfacalcidol resulted in improvement in hyperparathyroidism bone disease more frequently when compared with placebo (32% versus 3%) in predialysis patients.^(^ ^58^ ^)^ Similarly, adynamic bone disease developed more frequently in alfacalcidol‐treated patients (11% versus 6%). Mineralization parameters improved in the alfacalcidol group, whereas bone volume remained unchanged. Because these studies were performed more than 30 years ago, it is now difficult to apply these results to current practice.^(^ ^57^, ^58^ ^)^

Therapies with VDRAs also have side effects that may limit their use, in particular hypercalcemia and hyperphosphatemia due to increasing intestinal calcium and phosphate absorption.^(^ ^13^, ^22^, ^23^ ^)^ Two more recent RCTs comparing paricalcitol to placebo associated their usage with increased blood calcium levels but also failed to demonstrate improvements in cardiac structure and function (left ventricular mass index, diastolic and systolic functions).^(^ ^40^, ^59^ ^)^ Accordingly, the most recent KDIGO guidelines update suggested that VDRA usage should be limited for the management of severe and progressive SHPT in patients with CKD G4–G5 (not graded).^(^ ^34^ ^)^ The KDOQI Work Group also agreed with this statement.^(^ ^60^ ^)^ Still, there is ambiguity facing implementation of these new recommendations. In patients with CKD 5D, KDIGO guidelines suggest PTH‐lowering therapy with VDRAs (2B).^(^ ^34^ ^)^

Our systematic review and meta‐analysis have some limitations. First, only six studies reported fractures and the small sample sizes of included trials limited the power of the meta‐analysis. Second, BMD was inconsistently reported across studies and, thus, meta‐analysis could not be conducted for this outcome. Third, the high risk of bias of included trials limited our ability to conclude on the effect of VDRAs on bone outcomes. Finally, none of the studies, except one,^(^ ^39^ ^)^ were designed a priori to capture fracture event data. Thus, this may also have limited our ability to fully evaluate the effect of VDRAs on fracture risk.

Our study also has several strengths. We used a robust methodology according to the highest standards suggested by guidelines of the Cochrane Collaboration to ensure the validity and reproducibility of our results.^(^ ^29^ ^)^ We also included trial registers (ClinicalTrials.gov and ICTRP) in our search of literature as they are major sources of unpublished data. Furthermore, our search strategy was exhaustive and inclusive. Indeed, we used sensitivity‐maximizing filters that did not include bone outcomes as previously found in systematic reviews, which allowed us to screen a large number of potentially eligible citation. We also included predialysis and dialysis populations, which have commonly been evaluated separately in previous studies. Finally, our review gives an update on fracture risk in CKD patients treated with VDRAs.

In summary, we found no significant relationship between use of VDRAs and fracture risk in CKD patients with and without dialysis. Stratification of trials by age, sex, CKD type (dialysis versus no dialysis), baseline PTH levels, follow‐up duration, and overall risk of bias showed similar findings. Furthermore, as only three trials reported BMD at different sites and with different BMD measurements, a meta‐analysis could not be performed. Still, the absence of a significant or precise result should not be interpreted as evidence that VDRAs has no benefit in patients with CKD. This review highlights that the trials to date are limited in their power and ability to appropriately evaluate clinically meaningful outcomes. Thereby, future large RCTs specifically designed to determine the role of VDRAs on bone outcomes are highly needed.

## Disclosures

All authors state that they have no conflicts of interest.

5

### PEER REVIEW

The peer review history for this article is available at https://publons.com/publon/10.1002/jbm4.10611.

## Supporting information


**Supplemental Appendix S1.** Search strategies established for MEDLINE, EMBASE, CENTRAL, ClinicalTrials.gov, and ICTRP.
**Supplemental Appendix S2.** Funnel plot of included studies on the effect of vitamin D analogues on fracture risk.
**Supplemental Appendix S3.** PRISMA checklist.
