# Supplementary material for: Vitamin D Analogues and Fracture Risk in Chronic Kidney Disease: A Systematic Review and Meta‐Analysis of Randomized Controlled Trials
Source: JBMR Plus. 2022 Feb 21;6(4):e10611. doi: 10.1002/jbm4.10611 (PMC9009117; doi:10.1002/jbm4.10611)
Supplement: Supplementary file 1 — Supplemental Appendix S1. Search strategies established for MEDLINE, EMBASE, CENTRAL, ClinicalTrials.gov, and ICTRP. Supplemental Appendix S2. Funnel plot of included studies on the effect of vitamin D analogues on fracture risk. Supplemental Appendix S3. PRISMA checklist. [file JBM4-6-e10611-s001.docx]

**Vitamin D Analogues and Fracture Risk in Chronic Kidney Disease: A Systematic Review and Meta-Analysis of Randomized Controlled Trials**

Nada Khelifi^1,2^, Louis-Charles Desbiens MSc^1,2^, Aboubacar Sidibé MSc^1,2^, Fabrice Mac-Way MD^1,2^

^1^ CHU de Québec Research Center, Division of Nephrology, Endocrinology and Nephrology Axis, Quebec City, QC, Canada

^2^ Department of Medicine, Faculty of Medicine, Université Laval, Quebec City, QC, Canada

**LEGEND OF SUPPLEMENTARY APPENDIX**

**Supplementary appendix 1:** Search strategies established for MEDLINE, EMBASE, CENTRAL, ClinicalTrials.gov and ICTRP.

**Supplementary appendix 2:** Funnel plot of included studies on the effect of vitamin D analogues on fracture risk.

**Supplementary appendix 3:** PRISMA checklist

**SUPPLEMENTARY APPENDIX 1**

| MEDLINE’s Research strategy (OVID WEB) | |
| --- | --- |
| 1. | Kidney Diseases/ |
| 2. | Kidney Failure, Chronic/ |
| 3. | Renal Insufficiency/ |
| 4. | Renal Replacement Therapy/ or exp renal dialysis/ or exp hemofiltration/ |
| 5. | (chronic kidney or chronic renal).tw. |
| 6. | (CKD or CKF or CRD or CRF or ESKD or ESRD or ESKF or ESRF).tw. |
| 7. | (predialysis or dialysis).tw. |
| 8. | (haemodialysis or haemodialysis).tw. |
| 9. | (CAPD or CCPD or APD).tw. |
| 10. | 1 or 2 or 3 or 4 or 5 or 6 or 7 or 8 or 9 |
| 11. | exp Vitamin D/ |
| 12. | vitamin d.tw. |
| 13. | vitamin d2.tw. |
| 14. | vitamin d3.tw. |
| 15. | cholecalciferol.tw. |
| 16. | colecalciferol.tw. |
| 17. | hydroxyc?olecalciferol.tw. |
| 18. | dihydroxyc?olecalciferol |
| 19. | dihydrotachysterol.tw. |
| 20. | (maxicalcitol or maxacalcitol).tw. |
| 21. | oxacalcitriol.tw. |
| 22. | paricalcitol.tw |
| 23. | doxercalciferol.tw. |
| 24. | dihydroxyvitamin.tw. |
| 25. | hydroxyvitamin.tw. |
| 26. | falecalcitriol.tw. |
| 27. | calcitriol.tw. |
| 28. | alfacalcidol.tw. |
| 29. | alphacalcidol.tw. |
| 30. | calcifediol.tw. |
| 31. | calcipotriol.tw. |
| 32. | epicalcitriol.tw. |
| 33. | lexacalcitol.tw. |
| 34. | seocalcitol.tw. |
| 35. | tacalcitol |
| 36. | ergocalciferol.tw. |
| 37. | 11 or 12 or 13 or 14 or 15 or 16 or 17 or 18 or 19 or 20 or 21 or 22 or 23 or 24 or 25 or 26 or 27 or 28 or 29 or 30 or 31 or 32 or 33 or 34 or 35 or 36 |
| 38. | 10 and 37 |
| 39. | (randomized controlled trial or controlled clinical trial).pt. or randomized.ab. or placebo.ab. or drug therapy.fs. or randomly.ab. or trial.ab. or groups.ab. |
| 40. | exp animals/ not humans.sh. |
| 41. | 39 not 40 |
| 42. | 38 and 41 |

| EMBASE’s Research strategy | |
| --- | --- |
| 1. | 'kidney disease'/de |
| 2. | 'kidney failure'/de |
| 3. | 'chronic kidney failure'/de |
| 4. | 'hemodialysis'/exp |
| 5. | 'haemodialysis':ab,ti OR 'hemodialysis':ab,ti |
| 6. | 'dialysis':ab,ti |
| 7. | 'capd':ab,ti OR 'ccpd':ab,ti OR 'apd':ab,ti |
| 8. | 'predialysis':ab,ti |
| 9. | 'chronic renal':ab,ti OR 'chronic kidney':ab,ti |
| 10. | 'ckd':ab,ti OR 'ckf':ab,ti OR 'crd':ab,ti OR 'crf':ab,ti OR 'eskd':ab,ti OR 'esrd':ab,ti OR 'eskf':ab,ti OR 'esrf':ab,ti |
| 11. | #1 OR #2 OR #3 OR #4 OR #5 OR #6 OR #7 OR #8 OR #9 OR #10 |
| 12. | 'vitamin d'/exp |
| 13. | 'vitamin d':ab,ti |
| 14. | 'vitamin d2':ab,ti |
| 15. | 'vitamin d3':ab,ti |
| 16. | 'cholecalciferol':ab,ti |
| 17. | 'colecalciferol':ab,ti |
| 18. | 'hydroxyc?olecalciferol':ab,ti |
| 19. | 'dihydroxyc?olecalciferol':ab,ti |
| 20. | 'dihydrotachysterol':ab,ti |
| 21. | 'maxicalcitol':ab,ti OR 'maxacalcitol':ab,ti |
| 22. | 'oxacalcitriol':ab,ti |
| 23. | 'paricalcitol':ab,ti |
| 24. | 'doxercalciferol':ab,ti. |
| 25. | 'dihydroxyvitamin':ab,ti or hydroxycitamin':ab,ti |
| 26. | 'falecalcitriol':ab,ti |
| 27. | 'calcitriol':ab,ti |
| 28. | 'alfacalcidol':ab,ti |
| 29. | 'alphacalcidol':ab,ti |
| 30. | 'calcifediol':ab,ti |
| 31. | 'calcipotriol':ab,ti |
| 32. | 'epicalcitriol':ab,ti |
| 33. | 'lexacalcitol':ab,ti |
| 34. | 'seocalcitol':ab,ti |
| 35. | 'tacalcitol':ab,ti |
| 36. | 'ergocalciferol':ab,ti |
| 37. | #12 OR #13 OR #14 OR #15 OR #16 OR #17 OR #18 OR #19 OR #20 OR #21 OR #22 OR #23 OR #24 OR #25 OR #26 OR #27 OR #28 OR #29 OR #30 OR #31 OR #32 OR #33 OR #34 OR #35 OR #36 |
| 38. | 'random*':ab,ti OR 'clinical trial*':ab,ti,kw OR 'treatment outcome'/exp |
| 39. | #11 AND #37 AND #38 |

| CENTRAL’s Research strategy (Wiley Online Library) | |
| --- | --- |
| 1. | MeSH descriptor: [Kidney Diseases] this term only |
| 2. | MeSH descriptor: [Kidney Failure, Chronic] this term only |
| 3. | MeSH descriptor: [Renal Insufficiency] this term only |
| 4. | MeSH descriptor: [Renal Insufficiency] this term only |
| 5. | MeSH descriptor: [Renal Dialysis] this term only |
| 6. | MeSH descriptor: [Hemofiltration] this term only |
| 7. | (chronic next kidney):ti,ab,kw OR (chronic next renal):ti,ab,kw |
| 8. | (CKD):ti,ab,kw OR (CKF):ti,ab,kw OR (CRF):ti,ab,kw OR (ESKD):ti,ab,kw OR (ESRD):ti,ab,kw OR (ESKF):ti,ab,kw OR (ESRF):ti,ab,kw |
| 9. | (predialysis):ti,ab,kw OR (dialysis):ti,ab,kw |
| 10. | (haemodialysis):ti,ab,kw OR (hemodialysis):ti,ab,kw |
| 11. | (CAPD):ti,ab,kw OR (CCPD):ti,ab,kw OR (APD):ti,ab,kw |
| 12. | (#1 or #2 or #3 or #4 or #5 or #6 or #7 or #8 or #9 or #10 or #11) |
| 13. | MeSH descriptor: [Vitamin D] explode all trees |
| 14. | (vitamin next d):ti,ab,kw |
| 15. | (vitamin next d2):ti,ab,kw |
| 16. | (vitamin next d3):ti,ab,kw |
| 17. | (cholecalciferol):ti,ab,kw OR (colecalciferol):ti,ab,kw |
| 18. | (hydroxycholecalciferol):ti,ab,kw OR (hydroxycolecalciferol):ti,ab,kw |
| 19. | (dihydroxycholecalciferol):ti,ab,kw OR (dihydroxycolecalciferol):ti,ab,kw |
| 20. | (dihydrotachysterol):ti,ab,kw |
| 21. | (maxacalcitol):ti,ab,kw OR (maxicalcitol):ti,ab,kw |
| 22. | (oxacalcitriol):ti,ab,kw |
| 23. | (paricalcitol):ti,ab,kw |
| 24. | (doxercalciferol):ti,ab,kw |
| 25. | (doxercalciferol):ti,ab,kw |
| 26. | (hydroxyvitamin):ti,ab,kw |
| 27. | (falecalcitriol):ti,ab,kw |
| 28. | (calcitriol):ti,ab,kw |
| 29. | (alfacalcidol):ti,ab,kw |
| 30. | (alphacalcidol):ti,ab,kw |
| 31. | (calcifediol):ti,ab,kw |
| 32. | (calcifediol):ti,ab,kw |
| 33. | (epicalcitriol):ti,ab,kw |
| 34. | (lexacalcitol):ti,ab,kw |
| 35. | (seocalcitol):ti,ab,kw |
| 36. | (tacalcitol):ti,ab,kw |
| 37. | (tacalcitol):ti,ab,kw |
| 38. | (#13 or #14 or #15 or #16 or #17 or #18 #19 or #20 or #21 or #22 or #23 or #24 or #25 or #26 or #27 or #28 or #29 or #30 or #31 or #32 or #33 or #34 or #35 or #36 or #37) |
| 39. | (#12 and #38) |

| Clinicaltrials.gov | |
| --- | --- |
| 1. | renal OR kidney |
| 2. | vitamin d OR vitamin d2 OR vitamin d3 OR cholecalciferol OR colecalciferol OR hydroxycholecalciferol OR hydroxycolecalciferol OR dihydroxycholecalciferol OR dihydroxycolecalciferol OR dihydrotachysterol OR maxacalcitol OR maxicalcitol OR oxacalcitriol OR paricalcitol OR doxercalciferol OR dihydroxyvitamin OR hydroxyvitamin OR falecalcitriol OR calcitriol OR alfacalcidol OR alphacalcidol OR calcifediol OR calcipotriol OR epicalcitriol OR lexacalcitol OR seocalcitol OR tacalcitol OR ergocalciferol |
| 3. | #1 AND #2 |

| International Clinical Trials Registry Platform (ICTRP) | |
| --- | --- |
| 1. | renal OR kidney |
| 2. | vitamin d OR vitamin d2 OR vitamin d3 OR cholecalciferol OR colecalciferol OR hydroxycholecalciferol OR hydroxycolecalciferol OR dihydroxycholecalciferol OR dihydroxycolecalciferol OR dihydrotachysterol OR maxacalcitol OR maxicalcitol OR oxacalcitriol OR paricalcitol OR doxercalciferol OR dihydroxyvitamin OR hydroxyvitamin OR falecalcitriol OR calcitriol OR alfacalcidol OR alphacalcidol OR calcifediol OR calcipotriol OR epicalcitriol OR lexacalcitol OR seocalcitol OR tacalcitol OR ergocalciferol |
| 3. | #1 AND #2 |

**SUPPLEMENTARY APPENDIX 2**

Funnel plot of included studies on the effect of vitamin D analogues on fracture risk.


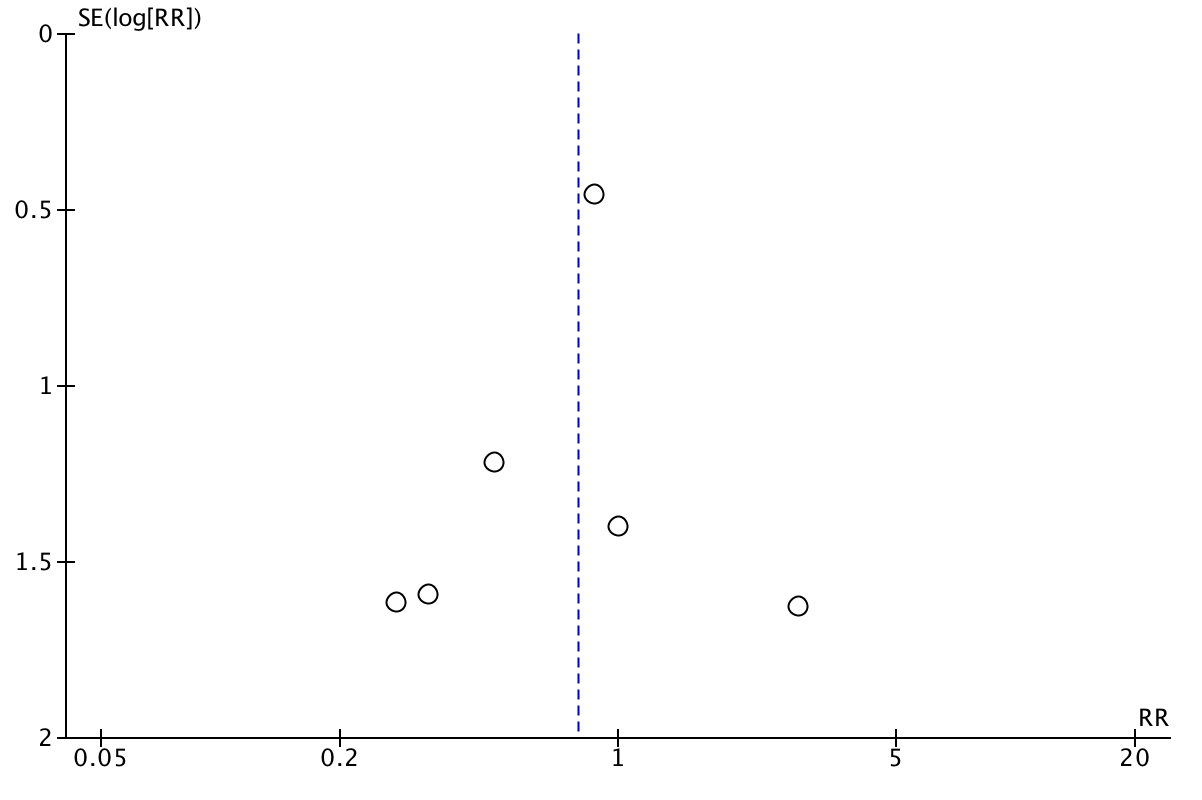


SE: Standard error; RR: Risk ratio.

**SUPPLEMENTARY APPENDIX 3**

| **Section/topic** | **#** | **Checklist item** | **Reported on page #** |
| --- | --- | --- | --- |
| **TITLE** | | |  |
| Title | 1 | Identify the report as a systematic review, meta-analysis, or both. | 1 |
| **ABSTRACT** | | |  |
| Structured summary | 2 | Provide a structured summary including, as applicable: background; objectives; data sources; study eligibility criteria, participants, and interventions; study appraisal and synthesis methods; results; limitations; conclusions and implications of key findings; systematic review registration number. | 3 |
| **INTRODUCTION** | | |  |
| Rationale | 3 | Describe the rationale for the review in the context of what is already known. | 5,6 |
| Objectives | 4 | Provide an explicit statement of questions being addressed with reference to participants, interventions, comparisons, outcomes, and study design (PICOS). | 5,6 |
| **METHODS** | | |  |
| Protocol and registration | 5 | Indicate if a review protocol exists, if and where it can be accessed (e.g., Web address), and, if available, provide registration information including registration number. | 7 |
| Eligibility criteria | 6 | Specify study characteristics (e.g., PICOS, length of follow-up) and report characteristics (e.g., years considered, language, publication status) used as criteria for eligibility, giving rationale. | 7, 8 |
| Information sources | 7 | Describe all information sources (e.g., databases with dates of coverage, contact with study authors to identify additional studies) in the search and date last searched. | 7 |
| Search | 8 | Present full electronic search strategy for at least one database, including any limits used, such that it could be repeated. | 7, Supplementary appendix 1 |
| Study selection | 9 | State the process for selecting studies (i.e., screening, eligibility, included in systematic review, and, if applicable, included in the meta-analysis). | 8 |
| Data collection process | 10 | Describe method of data extraction from reports (e.g., piloted forms, independently, in duplicate) and any processes for obtaining and confirming data from investigators. | 8,9 |
| Data items | 11 | List and define all variables for which data were sought (e.g., PICOS, funding sources) and any assumptions and simplifications made. | 9 |
| Risk of bias in individual studies | 12 | Describe methods used for assessing risk of bias of individual studies (including specification of whether this was done at the study or outcome level), and how this information is to be used in any data synthesis. | 9 |
| Summary measures | 13 | State the principal summary measures (e.g., risk ratio, difference in means). | 9,10 |
| Synthesis of results | 14 | Describe the methods of handling data and combining results of studies, if done, including measures of consistency (e.g., I^2^) for each meta-analysis. | 9,10 |

| **Section/topic** | **#** | **Checklist item** | **Reported on page #** |
| --- | --- | --- | --- |
| Risk of bias across studies | 15 | Specify any assessment of risk of bias that may affect the cumulative evidence (e.g., publication bias, selective reporting within studies). | 10 |
| Additional analyses | 16 | Describe methods of additional analyses (e.g., sensitivity or subgroup analyses, meta-regression), if done, indicating which were pre-specified. | 10 |
| **RESULTS** | | |  |
| Study selection | 17 | Give numbers of studies screened, assessed for eligibility, and included in the review, with reasons for exclusions at each stage, ideally with a flow diagram. | 11, 32 |
| Study characteristics | 18 | For each study, present characteristics for which data were extracted (e.g., study size, PICOS, follow-up period) and provide the citations. | 11, 12, 26,27 |
| Risk of bias within studies | 19 | Present data on risk of bias of each study and, if available, any outcome level assessment (see item 12). | 13, 29, 30 |
| Results of individual studies | 20 | For all outcomes considered (benefits or harms), present, for each study: (a) simple summary data for each intervention group (b) effect estimates and confidence intervals, ideally with a forest plot. | 12, 28, 33-35 |
| Synthesis of results | 21 | Present results of each meta-analysis done, including confidence intervals and measures of consistency. | 12, 28, 33-35 |
| Risk of bias across studies | 22 | Present results of any assessment of risk of bias across studies (see Item 15). | 12, Supplementary appendix 2 |
| Additional analysis | 23 | Give results of additional analyses, if done (e.g., sensitivity or subgroup analyses, meta-regression [see Item 16]). | 12, 28 |
| **DISCUSSION** | | |  |
| Summary of evidence | 24 | Summarize the main findings including the strength of evidence for each main outcome; consider their relevance to key groups (e.g., healthcare providers, users, and policy makers). | 14-16 |
| Limitations | 25 | Discuss limitations at study and outcome level (e.g., risk of bias), and at review-level (e.g., incomplete retrieval of identified research, reporting bias). | 17 |
| Conclusions | 26 | Provide a general interpretation of the results in the context of other evidence, and implications for future research. | 18 |
| **FUNDING** | | |  |
| Funding | 27 | Describe sources of funding for the systematic review and other support (e.g., supply of data); role of funders for the systematic review. | 19 |
